# Prenatal Perfluorooctanoic Acid (PFOA) Exposure Is Associated With Lower Infant Birthweight Within the MADRES Pregnancy Cohort

**DOI:** 10.3389/fepid.2022.934715

**Published:** 2022-07-13

**Authors:** Alicia K. Peterson, Sandrah P. Eckel, Rima Habre, Tingyu Yang, Dema Faham, Shohreh F. Farzan, Brendan H. Grubbs, Kurunthachalam Kannan, Morgan Robinson, Deborah Lerner, Laila A. Al-Marayati, Daphne K. Walker, Edward G. Grant, Theresa M. Bastain, Carrie V. Breton

**Affiliations:** ^1^Department of Population and Public Health Sciences, Keck School of Medicine, University of Southern California, Los Angeles, CA, United States; ^2^Department of Obstetrics and Gynecology, Keck School of Medicine, University of Southern California, Los Angeles, CA, United States; ^3^Department of Pediatrics, New York University School of Medicine, New York, NY, United States; ^4^Eisner Pediatric and Family Medical Center, Eisner Health, Los Angeles, CA, United States; ^5^Department of Radiology, Keck School of Medicine, University of Southern California, Los Angeles, CA, United States

**Keywords:** PFAS, perfluorooctanoic acid (PFOA), birthweight, health disparities, pregnancy

## Abstract

**Introduction:**

Perfluoroalkyl and polyfluoroalkyl substances (PFAS) are persistent synthetic chemicals found in household products that can cross the placenta during pregnancy. We investigated whether PFAS exposure during pregnancy was associated with infant birth outcomes in a predominantly urban Hispanic population.

**Methods:**

Serum concentrations of perfluorooctanoic acid (PFOA), perfluorooctanesulfonic acid (PFOS), perfluorohexanesulfonic acid (PFHxS), perfluorononanoic acid (PFNA), and perfluorodecanoic acid (PFDA) were measured in 342 prenatal biospecimens (mean gestational age: 21 ± 9 weeks) from participants in the ongoing Maternal And Developmental Risks from Environmental and Social Stressors (MADRES) cohort. PFAS compounds were modeled continuously or categorically, depending on the percentage of samples detected. The birth outcomes assessed were birthweight, gestational age at birth, and birthweight for gestational age (BW-for-GA) z-scores that accounted for parity or infant sex. Single pollutant and multipollutant linear regression models were performed to evaluate associations between PFAS exposures and birth outcomes, adjusting for sociodemographic, perinatal, and study design covariates.

**Results:**

Maternal participants (n = 342) were on average 29 ± 6 years old at study entry and were predominantly Hispanic (76%). Infants were born at a mean of 39 ± 2 weeks of gestation and weighed on average 3,278 ± 522 g. PFOS and PFHxS were detected in 100% of the samples while PFNA, PFOA, and PFDA were detected in 70%, 65%, and 57% of the samples, respectively. PFAS levels were generally lower in this cohort than in comparable cohorts. Women with detected levels of PFOA during pregnancy had infants weighing on average 119.7 g less (95% CI −216.7, −22.7) than women with undetected levels of PFOA in adjusted single pollutant models. PFOA results were also statistically significant in BW-for-GA z-score models that were specific for sex or parity. In models that were mutually adjusted for five detected PFAS compounds, PFOA results remained comparable; however, the association was only significant in BW-for-GA z-scores that were specific for parity (β = −0.3; 95% CI −0.6, −0.01). We found no significant adjusted associations with the remaining PFAS concentrations and the birth outcomes assessed.

**Conclusion:**

Prenatal exposure to PFOA was associated with lower birthweight in infants, suggesting that exposure to these chemicals during critical periods of development might have important implications for children's health.

## Introduction

Perfluoroalkyl and polyfluoroalkyl substances (PFAS) are a class of ubiquitous synthetic chemicals that have bioaccumulation properties with a long half-life and are commonly found in household products due to their water- and stain-resistant qualities. Across the world, these persistent chemicals have been found in humans, marine and land animals, and soil and water within the environment (1). Within this class of chemicals, most research has focused on perfluorooctanoic acid (PFOA) and perfluorooctanesulfonic acid (PFOS); however, there are more than 4,700 compounds that are considered PFAS (2). Animal and *in vitro* studies have found that PFOA and PFOS have a toxic effect on the liver, kidney, reproductive system, cardiovascular system, and endocrine system (3–6). Prenatal exposure to PFOA and PFOS has also consistently led to lower birthweight and offspring mortality in mice and rats (7, 8). Although PFOA, PFOS, and PFAS with similar chemical structures have been phased out of manufacturing in the USA (9), products containing them can still be imported. In addition, due to their long half-lives [PFOA 4 years and PFOS 5 years (10)], the substances are still commonly found in the blood serum of the US population, even in children born after the phase-out in 2002 (11, 12). A wide range of items contain PFAS, such as nonstick cookware, shampoos, waterproof clothing, fire-fighting foam, electronics, and fast-food packaging (2, 13, 14). The most common exposure routes of PFAS to humans include ingestion through drinking water or contaminated food (i.e., fish or food stored in PFAS-containing packaging transferred into food), inhalation of indoor air and dust, and occupational exposure through inhalation or dermal contact (14–19).

Exposure to these substances may be particularly critical during fetal development due to their ability to cross the placenta during pregnancy (20). The placenta has been shown to be vulnerable to environmental insults, to be a common target of PFAS, and to accumulate across gestation *via* the maternal circulation (20–22). No consensus has been reached on the exact biological mechanism by which PFAS influence fetal growth; however, proposed hypotheses include impacts on bone development, maternal hormone disruption—particularly sex and thyroid hormones—and adverse effects on placental development and function (23–30). Several recent meta-analyses and systematic literature reviews suggest that prenatal exposure to PFOA and PFOS is associated with lower infant birthweight (8, 31–34). However, many individual studies have failed to detect significant associations. Additionally, several biases have been discussed, especially for studies that measure concentrations late in pregnancy or at birth, due to biological and physiological changes that occur in later pregnancy, such as increased glomerular filtration (33).

Epidemiological studies have also found statistically significant associations between some PFAS compounds, such as perfluorohexanesulfonic acid (PFHxS), perfluorononanoic acid (PFNA), and perfluorodecanoic acid (PFDA) with infant birthweight (35–40); however, these relationships have been assessed in far fewer studies than in studies with PFOA and PFOS. Many recently published studies analyzing these associations used specimens collected in the early 2000s, which likely do not represent current exposure levels due to the phase outs and use of replacement compounds that have occurred in recent decades (37, 38, 40, 41).

A better understanding of the association between prenatal PFAS exposure, particularly compounds other than PFOA and PFOS, and infant birthweight is important as low birthweight, defined as <2,500 g, is a strong predictor of childhood obesity and subsequent metabolic health morbidities, such as type 2 diabetes later in life (42–44). In the USA, pregnant persons who identify as Black or Hispanic have a higher rate of infants born with low birthweight compared to their non-Hispanic white counterparts (45). PFAS concentration levels also differ by race and ethnicity. The National Health and Nutrition Examination Survey (NHANES) found that non-Hispanic whites have higher concentration levels with reference to Blacks or Hispanics (11). However, few epidemiologic studies have investigated the influence of PFAS exposure on birth outcomes among racially and ethnically diverse populations, specifically within Hispanic populations.

The objective of this study was to determine if maternal serum PFAS concentrations measured in pregnancy were associated with infant birthweight and birthweight for gestational age (BW-for-GA) *z*-scores in a prospective pregnancy cohort representing a structurally marginalized population. Additionally, we examined the relationship between prenatal PFAS serum concentrations and gestational age at birth.

## Methods

### Study Sample

Participants included in this study were drawn from the ongoing Maternal And Developmental Risks from Environmental and Social Stressors (MADRES) pregnancy cohort, which is predominately comprised of Hispanic participants and their children residing in urban Los Angeles, CA, USA. Methods of the MADRES study and protocol have been described previously (46). Briefly, participants were enrolled during pregnancy at four prenatal clinic sites. These included two community health clinics, one county hospital prenatal clinic, and one private obstetrics and gynecology practice. Eligibility for participants at the time of recruitment included: (1) <30 weeks pregnant (2) at least 18 years of age; and (3) being fluent in English or Spanish. Exclusion criteria for the study included: (1) multiple gestation; (2) having a physical, mental, or cognitive disability that would prevent participation or ability to provide consent; (3) being currently incarcerated; or (4) HIV positive status. At study entry, informed consent and The Health Insurance Portability and Accountability Act (HIPAA) authorization were obtained for medical record abstraction from all participants. The University of Southern California's Institutional Review Board approved all study procedures.

Blood serum samples were collected from 359 MADRES participants between December 2015 and February 2019, and PFAS concentrations were measured in Spring 2019. Of the 359 participants with PFAS measurements, 347 participants had available data on infant birthweight. We removed five participants who withdrew from the study prior to birth (two study dropouts and three miscarriages) as well as seven participants whose infants lacked birthweight. Additionally, five participants were removed due to missing race/ethnicity. A total of 342 mother–infant dyads with PFAS analyte concentrations, birth outcomes data, and key covariate information were included in the current study.

### Exposure Assessment of Prenatal PFAS Concentrations

Blood samples were collected during pregnancy using red top 10-ml serum tubes at an in-person study visit (median gestational age: 19.1 weeks; range 5.7–38.3 weeks). Samples were processed, and serum was aliquoted into appropriately labeled 0.5-ml cryovials before storage in a −80°C freezer prior to shipment to the analytical laboratory.

Samples were sent to the Wadsworth Center's Human Health Exposure Assessment Resource (WC-HHEAR) laboratory at the NYU Langone Medical Center (Dr. Kannan's laboratory) for analysis. A total of 14 PFAS, namely, PFHxS, PFOS, PFOA, PFNA, PFDA, perfluorobutanesulfonic acid (PFBS), perfluoroheptanoic acid (PFHPA), perfluroundecanoic acid (PFUNDA), perfluorododecanoic (PFDODA), perfluorooctanesulfonamide (PFOSA), n-ethyl perfluorooctane sulfonamido acetic acid (NETFOSAA), n-methyl perfluorooctane sulfonamido acetic acid (NMFOSAA), perfluoro-n-pentanoic acid (PFPEA), and perfluorohexanoic acid (PFHxA) were analyzed. The method for the analysis of 14 PFAS in serum has been described elsewhere (47). In brief, serum samples (0.25 ml) were aliquoted into 15-ml polypropylene (PP) tubes and spiked with 5 ng of ^13^C-labeled internal standard (IS) mixture and 0.7 ml of 1% ammonium formate (w/v) in methanol (MeOH). The mixture was centrifuged for 5 min at 5,000 rpm, and the supernatant was collected and passed through the Hybrid-SPE cartridge (Phospholipid, 30 mg, 1 cc, Sigma-Aldrich, St. Louis, MO, USA). Cartridges were conditioned with 1 ml of MeOH containing 1% ammonium formate (w/v). Samples were eluted through the cartridge and collected in a PP tube for the LC-MS/MS analysis.

Target analytes were quantified by the isotopic dilution method, and a 12-point calibration (at concentrations ranging from 0.02 to 100 ng/ml) with the regression coefficient of ≥ 0.999 was used. A pure solvent (MeOH) and a mid-point calibration standard (5 ng/ml) were injected after every 10 samples to check for the carryover of target chemicals and instrumental drift in sensitivity. Several procedural blanks were analyzed to monitor for contamination that potentially arise from reagents and materials used in sample preparation steps. For each batch of 100 samples, five duplicates of procedural blanks and QC spiked samples (water spiked with native standards at 5 ng for all analytes and IS) were processed. Duplicates of Standard Reference Material (SRM1958, NIST, Gaithersburg, MD, USA; IS spiked) containing certified values for PFHxS, PFOS, PFOA, and PFNA were analyzed. Trace levels of NETFOSAA (0.001–0.047 ng/ml) and NMFOSAA (0.002–0.012 ng/ml) were found in procedural blanks, and the concentrations of these chemicals in samples were subtracted from blank values. Spiked sample and SRM1958 recoveries were in the range of 81.5–112% [Relative standard deviation (RSD): ±4.3–8.7%] and 86.7–111% (RSD: ±2.1–6.8%), respectively. The limit of detection (LOD) of target analytes ranged from 0.02 to 0.05 ng/ml. Five of the 14 PFAS were detected in at least 50% of MADRES participant samples and were included in subsequent data analysis. These included PFOS (100% detected), PFHxS (100% detected), PFNA (70% detected), PFOA (65% detected), and PFDA (57% detected).

### Birth Outcomes

Infant birthweight was directly abstracted from electronic medical records (EMR) for all but one participant (99.7%). The remaining participant did not have a physician measurement of birthweight recorded on the EMR, and birthweight was obtained from the participant *via* an interviewer-administered questionnaire with a MADRES staff member 7–14 days post-birth. Gestational age at birth was calculated and standardized using a hierarchy of methods (48). A first trimester (<14 weeks of gestation) ultrasound measurement of crown-rump length was deemed ideal and was used if available (59.7%). If unavailable, a second trimester (<28 weeks of gestation) ultrasound measurement of fetal biparietal diameter was used (26.9%). If measurements from an early ultrasound were unavailable, gestational age at birth was calculated based on a physician's best clinical estimate from EMR (13.2%). If none of the above mentioned measurements were available, gestational age was calculated from the estimated last menstrual period (<1%). We evaluated the birthweight by modeling it continuously as well as by calculating sex-specific or parity specific BW-for-GW *z*-scores based on Aris et al. (49). These *z*-scores reflect the current sociodemographic composition of the USA using 2017 US natality files from obstetric estimated gestational age on singleton births, which replaced previous BW-for-GA *z*-scores estimated using self-reported last menstrual period (49).

### Covariates

Potential covariates were identified *a priori* from the literature and included participant demographics, aspects related to study design, and pregnancy- and birth-related variables. Study design variables were the study recruitment site and gestational age at the time of blood serum collection.

Participant demographic covariates included race/ethnicity, country of birth, the highest attained education, annual household income, participant's age at the time of study enrollment, any reported personal smoking during pregnancy, and pre-pregnancy body mass index (BMI: kg/m^2^). These variables were self-reported *via* interviewer-administered questionnaires in English or Spanish. Pre-pregnancy BMI was calculated using self-reported pre-pregnancy weight and standing height at the first study visit (<30 weeks of gestation) measured by a study staff member *via* a stadiometer (perspectives enterprises model PE-AIM-101).

Pregnancy-related covariates included parity, which was self-reported *via* an interviewer-administered questionnaire at the first study visit, infant sex abstracted from EMR (96.8%) or self-reported from the participant (3.2%), and the best available gestational age at the time of birth for continuous birthweight models. In addition, information on maternal seafood consumption during the pregnancy was obtained *via* an interviewer-administered questionnaire in English or Spanish during the third trimester. Participants were asked if they had ever consumed any of the following types of seafood during the pregnancy: fish sticks, fresh oily fish, other fresh fish, canned tuna, shellfish, or fried shellfish. A combined variable was then created with four categories: never (62.6%), monthly (16.1%), at least weekly (12.3%), or unknown fish consumption status (9.0%).

DAGitty was used to create a directed acyclic graph (DAG) using the above mentioned covariates to assess relationships (Supplementary Figure S1) (50). Minimal sufficient adjustment sets for estimating the total effect of prenatal PFAS exposure on birthweight were child's sex, country of birth, fish consumption, gestational age at the time of blood sampling, household income, maternal age, maternal education, parity, race/ethnicity, and pre-pregnancy BMI. Continuous birthweight models were adjusted for child's sex (male, female), country of birth (USA, other, missing indicator), fish consumption (never, monthly, at least weekly, missing indicator), gestational age at birth (weeks), gestational age at the time of blood sampling (weeks), household income (<30 k, 30–99 k, >100 k, or reported “Do Not Know”), maternal education (high school or less, some/completed college, or some graduate training), parity (first born, second or more born, missing indicator), race/ethnicity (Hispanic, non-Hispanic white, non-Hispanic Black, non-Hispanic other), pre-pregnancy BMI (kg/m^2^), maternal age at study recruitment (years), and recruitment site. BW-for-GA *z*-score models included the same covariates except for gestational age at birth. Sex specific BW-for-GA *z*-score models removed infant sex while parity-specific BW-for-GA *z*-score models removed parity.

### Statistical Analysis

Bivariate analyses were conducted with covariates to assess their relationships with the PFAS compounds ran continuously as well as with birthweight and gestational age at birth. Due to the right skewed distributions of the PFAS compounds, Kruskal–Wallis one way analysis of variance (ANOVA) tests were used to assess relationships with categorical variables including education, household income, country of birth, race/ethnicity, parity, fish consumption, and recruitment site. Spearman correlations were performed for associations between PFAS compounds and continuous variables including participant's age, pre-pregnancy BMI, and gestational age at the time of blood serum collection. Spearman correlations were also used to assess the correlation between the five compounds. For PFAS samples that were below the LOD, values were imputed using the equation of LOD/2 (51).

We used multiple linear regression models to assess the relationships between PFAS concentrations and birthweight and BW-for-GA *z*-scores that were parity or sex specific. Depending on the percentage of the samples detected for each PFAS compound, we either modeled the exposure as a continuous exposure or categorical exposure (e.g., detected/non-detected or quartiles of exposure). Because PFHxS and PFOS were detected in 100% of samples, we log transformed these exposures and modeled them on the continuous scale. We also modeled these two compounds categorically by quartiles. PFOA, PFNA, and PFDA were detected in 57–70% of samples; therefore, we modeled these exposures categorically (detected vs. non-detected). Additionally, multipollutant models were carried out to mutually for the five PFAS. Single pollutant linear regression models and multipollutant models had no evidence of multicollinearity based on the variance inflation factor (VIF) (all individual variables VIF < 10, mean VIF < 2), and no influential points were identified (Cook's D < 1). Modeling assumptions for linear regression were checked and met for all models.

A statistical interaction between each PFAS compound and infant sex was tested within single pollutant models, and models were stratified by infant sex to assess for effect modification. A sensitivity analysis was carried out by excluding participants who had gestational diabetes mellitus (GDM) (*n* = 33), gestational hypertension (*n* = 26), or preeclampsia/eclampsia (*n* = 25) recorded on their medical record, although some women overlapped with GDM and a gestational hypertension disorder (*n* = 3). Lastly, an additional sensitivity analysis was carried out where models were restricted to only participants who had prenatal PFAS concentrations measured in blood samples prior to 30 weeks of gestation (*n* = 239).

Adjusted linear regression models were also performed to assess the relationship between prenatal PFAS blood serum and gestational age at birth to investigate whether gestational age was a biological intermediate between prenatal PFAS and infant birthweight (31, 52).

The significance level for all analyses was set at an alpha of 0.05, and all data were analyzed using SAS version 9.4 (SAS Institute, Inc., Cary, NC, USA).

## Results

### Participant Characteristics

Participant and infant characteristics are reported in Table 1. Participants were on average 29 ± 6 years old at study recruitment, 76% were Hispanic, the majority (53%) had a high school diploma or less and had a mean pre-pregnancy BMI of 28 ± 6 kg/m^2^. Their infants were born at a mean of 39 ± 2 weeks of gestation and weighed on average 3,278 ± 522 g at birth. The majority of infants were at least the second born child (62%). Maternal smoking during any point in pregnancy was low in this population (*n* = 6).

**Table 1:** Demographics of 342 mother–infant dyads.

**Characteristic**	***N* (%) or Mean (SD)**
**Maternal**	
Age (years)	29.3 (6.0)
**Race/Ethnicity**	
Hispanic	260 (76.0%)
Non-Hispanic black	37 (10.8%)
Non-Hispanic white	30 (8.8%)
Non-Hispanic other	15 (4.4%)
**Country of birth**	
USA	175 (51.2%)
Other	157 (45.9%)
Unknown	10 (2.9%)
**Household income**	
<$50,000	165 (48.2%)
$50,000-$99,999	65 (19.0%)
>$100,000	28 (8.2%)
Reported “Do not Know”	84 (24.6%)
**Education**	
Completed high school or less	181 (52.9%)
Some college or completed college	133 (38.9%)
Some graduate training	28 (8.2%)
Pre-Pregnancy BMI (kg/m^2^)	28.2 (6.1)
Any personal smoking during pregnancy	6 (1.8%)
**Infant**	
Gestational age at birth (weeks)	38.9 (1.8)
Birthweight (g)	3,278.6 (522.2)
Premature (<37 weeks)	36 (10.5%)
Low birth weight (<2,500 g)	17 (5.0%)
Male	176 (51.5%)
**Birth order of child**	
First born	119 (34.8%)
Second or more born	212 (62.0%)
Unknown	11 (3.2%)

### PFAS Analytes

**Figure 1:**
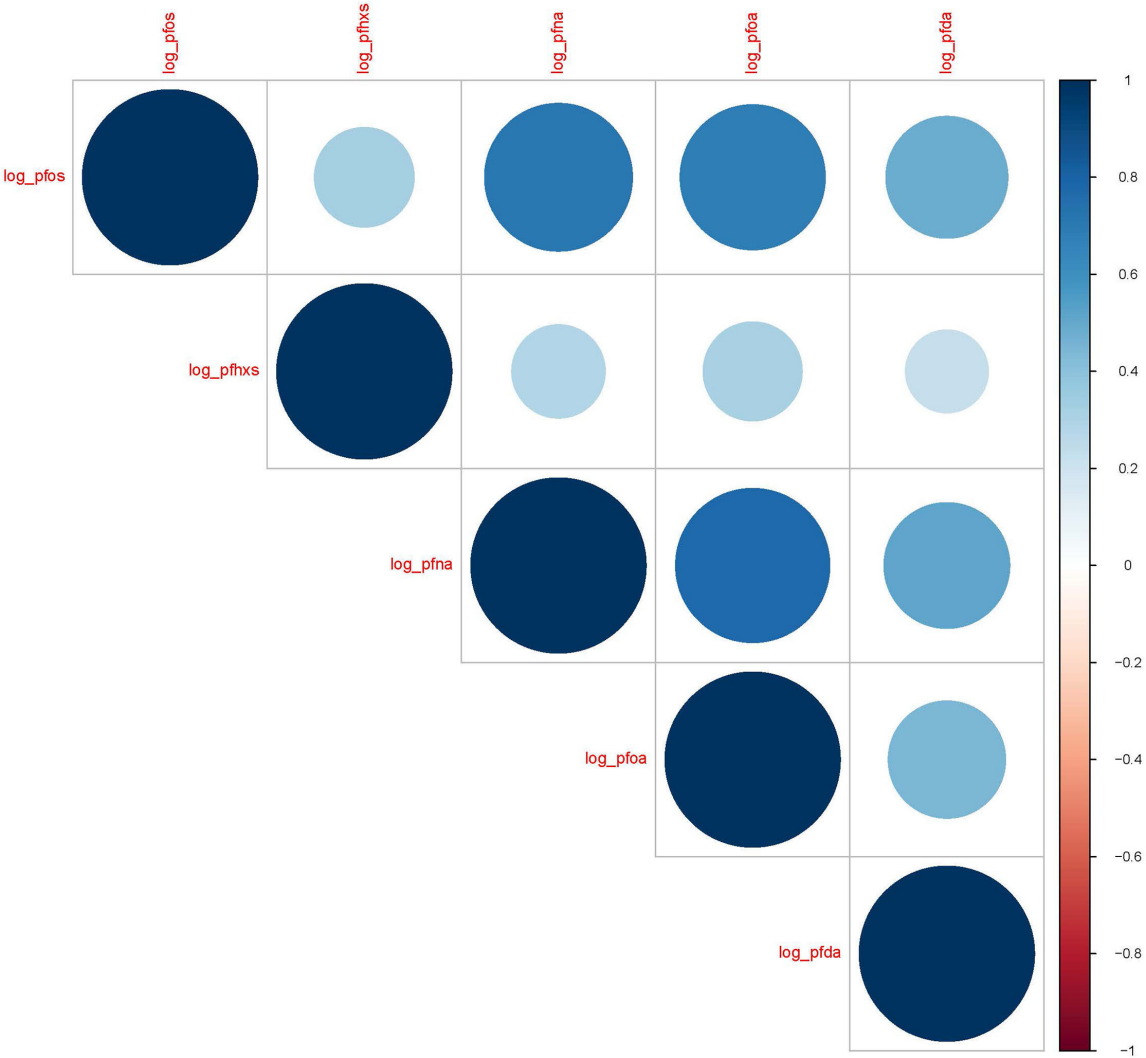
Spearman correlation coefficients for natural log transformed PFAS (ng/mL) in maternal blood serum (*N* = 342). *p* < 0.0001 for all correlations.

Five PFAS analytes had > 50% of samples above the LOD (PFOS 100%, PFHxS 100%, PFNA 70%, PFOA 65%, and PFDA 57%). The remaining analytes had 17% or less of samples that were above the LOD and were excluded from this analysis. The median concentrations were the highest for PFOS (1.34 ng/ml) followed by PFHxS (1.09 ng/ml). Distributions for all measured analytes are shown in Table 2. The natural log-transformed PFAS concentrations were also significantly positively correlated with each other and are shown in Figure 1 (Spearman's *R* = 0.21 to 0.78, *p* < 0.0001 for all correlations). PFAS concentration levels seen within the MADRES cohort, with the exception of PFHxS, were lower than the levels reported in comparable cohorts (53–55).

**Table 2:** Distribution of perfluoroalkyl and polyfluoroalkyl substances (PFAS) (ng/ml) concentrations in maternal blood serum (*n* = 342).

**Analyte**	**Abbreviation**	**LOD (ng/ml)**	**% Above LOD**	**Min**	**Q 1**	**Q 2**	**Q 3**	**Max**
Perfluorooctanesulfonic acid	PFOS	0.02	100%	0.09	0.97	1.34	1.86	10.36
Perfluorohexanesulfonic acid	PFHxS	0.02	100%	0.36	0.79	1.09	1.47	4.1
Perfluorononanoic acid	PFNA	0.02	70%	ND	ND	0.07	0.19	1.51
Perfluorooctanoic acid	PFOA	0.035	65%	ND	ND	0.12	0.39	4.93
Perfluorodecanoic acid	PFDA	0.035	57%	ND	ND	0.04	0.09	2.32
Perfluoro-n-pentanoic acid	PFPEA	0.05	17%	ND	ND	ND	ND	1.19
Ethyl Perfluorooctane sulfonamido acetic acid	NETFOSAA	0.02	12%	ND	ND	ND	ND	0.12
N-methyl Perfluorooctane sulfonamido acetic acid	NMFOSAA	0.02	10%	ND	ND	ND	ND	0.38
Perfluroundecanoic acid	PFUNDA	0.02	9%	ND	ND	ND	ND	0.72
Perfluorohexanoic acid	PFHxA	0.05	1%	ND	ND	ND	ND	0.11
Perfluorobutanesulfonic acid	PFBS	0.02	1%	ND	ND	ND	ND	0.08
Perfluorododecanoic acid	PFDODA	0.035	1%	ND	ND	ND	ND	0.94
Perfluorooctanesulfonamide	PFOSA	0.02	0%	ND	ND	ND	ND	ND
Perfluoroheptanoic acid	PFHPA	0.05	0%	ND	ND	ND	ND	ND

*ND, not detected; Q1, Quartile 1 (25th Percentile); Q2, Quartile 2 (50th Percentile); Q3, Quartile 3 (75th Percentile)*.

Median levels of PFAS by demographic characteristics are shown in Supplementary Table S1. Significant differences were observed in median PFOA, PFNA, PFHxS, and PFOS by education levels, and participants with at least some graduate training had the highest levels. These trends were consistent with household income with median PFOA, PFNA, PFHxS, and PFOS differing across income bracket and those making at least $100,000 annually having the highest levels. Significant differences in median PFOA, PFNA, PFHxS, and PFOS were observed by maternal race with non-Hispanic white participants having the highest levels. Additionally, there were significant differences in median PFOA, PFDA, PFNA, PFHxS, and PFOS by the parity status with participants pregnant with their first child having the highest levels. Median PFNA, PFHxS, and PFOS significantly differed in fish consumption habits during pregnancy with those who consumed fish at least weekly having the highest levels. Significant differences in median PFOS and PFHxS were observed by country of birth, with participants born outside of the USA having lower levels. All PFAS concentrations were significantly inversely associated with pre-pregnancy BMI (Spearman's *R* = −0.29 to −0.13, *p* < 0.02 for all correlations) while no relationships were observed with PFAS and maternal age (*p* > 0.14 for all correlations).

### Single Pollutant Linear Regression Models

No significant associations were observed with any PFAS compound and gestational age at birth in univariate or adjusted models (Supplementary Table S2). Therefore, we concluded that gestational age was not acting as a mediator of the association between prenatal PFAS exposure and birthweight within the MADRES cohort. We included gestational age at birth as a covariate in continuous birthweight models. The results for single prenatal PFAS exposures with continuous infant birthweight and BW-for-GA *z*-scores that were parity or sex specific are shown in Table 3. Women with detected levels of PFOA (β = −119.7 g; 95% CI −216.7, −22.7) and PFNA (β = −105.8 g; 95% CI −206.3, −5.3) had infants with significantly lower birthweight on average compared to women without detected levels. No significant associations were seen with the remaining PFAS compounds and infant birthweight, although non-significant inverse relationships were seen with PFOS and PFDA and a non-significant positive relationship was seen with PFHxS. The results for all PFAS were similar in BW-for-GA *z*-score models compared to continuous birthweight models, although only PFOA remained significant in models with sex-specific BW-for-GA *z*-scores (β = −0.3; 95% CI −0.5, −0.1) and parity-specific BW-for-GA *z*-scores (β = −0.3; 95% CI −0.5, −0.1). No evidence was found for a statistical interaction between any PFAS compound and infant sex (all interaction *p* > 0.29), and there were no significant differences in stratified models by sex (data not shown).

**Table 3:** Adjusted single PFAS exposure models with continuous birthweight and sex- or parity-specific birthweight for gestational age (BW-for-GA) *z*-scores (*n* = 342).

**Model**	**Birthweight^a^ β (95% CI)**	**BW-for-GA z-scores, sex^b^** **β (95% CI)**	**BW-for-GA z-scores, parity^c^ β (95% CI)**
PFOS	−37.2^§^ (−123.9, 49.6)	−0.1^§^ (−0.3, 0.1)	−0.1^§^ (−0.3, 0.2)
<0.97 ng/ml	REF	REF	REF
0.97–1.33 ng/ml	−41.5 (−167.9, 85.0)	−0.1 (−0.5, 0.2)	−0.2 (−0.5, 0.2)
1.34–1.85 ng/ml	−51.4 (−178.0, 75.2)	−0.1 (−0.4, 0.2)	−0.1 (−0.4, 0.2)
≥1.86 ng/ml	−53.9 (−188.8, 80.9)	−0.1 (0.5, 0.2)	−0.1 (−0.5, 0.2)
PFHxS	49.7^§^ (−78.7, 178.2)	−0.1^§^ (−0.2, 0.5)	−0.2^§^ (−0.2, 0.5)
<0.79 ng/ml	REF	REF	REF
0.79–1.08 ng/ml	81.6 (−44.1, 207.4)	0.2 (−0.1, 0.7)	0.2 (−0.1, 0.6)
1.09–1.46 ng/ml	24.4 (−109.6, 158.3)	0.1 (−0.3, 0.4)	0.1 (−0.2, 0.4)
≥1.47 ng/ml	118.1 (−32.3, 267.5)	0.3 (−0.1, 0.7)	0.3 (−0.1, 0.7)
PFOA			
≤LOD	REF	REF	REF
>LOD	−119.7 (−216.7, −22.7)^*^	−0.3 (−0.5, −0.1)^*^	−0.3 (−0.5, -0.1)^*^
PFNA			
≤LOD	REF	REF	REF
>LOD	−105.8 (−206.3, −5.3)^*^	−0.2 (−0.5, 0.01)	−0.2 (−0.5, 0.01)
PFDA			
≤LOD	REF	REF	REF
>LOD	−55.4 (−145.1, 34.3)	−0.2 (−0.4, 0.1)	−0.2 (−0.4, 0.1)

*CI, Confidence interval; PFHxS, Perfluorohexanesulfonic acid; PFOS, Perfluorooctanesulfonic acid; PFNA, Perfluorononanoic acid; PFOA, Perfluorooctanoic acid; PFDA, Perfluorodecanoic acid. Reference group: Samples <LOD; PFNA LOD, 0.02 ng/ml, PFDA LOD, 0.035 ng/ml, PFOA LOD, 0.035 ng/ml*.

a *Adjusted for maternal race, maternal age at study recruitment, country of birth, maternal education, maternal household income, recruitment site, GA at time of blood sample, pre–pregnancy body mass index (BMI), pregnancy fish consumption, GA at birth, parity and infant sex*.

b *Adjusted for maternal race, maternal age at study recruitment, country of birth, maternal education, maternal household income, recruitment site, GA at time of blood sample, pre–pregnancy BMI, pregnancy fish consumption, and parity*.

c *Adjusted for maternal race, maternal age at study recruitment, country of birth, maternal education, maternal household income, recruitment site, GA at time of blood sample, pre–pregnancy BMI, pregnancy fish consumption, and infant sex, n, 331*.

§ *β-Estimate interpreted as per natural log increase*.

* *p < 0.05*.

### Multipollutant Linear Regression Models

In models that were mutually adjusted for all five PFAS compounds, the effect estimate was slightly attenuated from independent models for the detected/non-detected PFOA and continuous birthweight and the detected/non-detected PFOA was no longer statistically significant (β = −108.8 g; 95% CI −229.9, 12.3). In contrast, the effect estimates for the detected/non-detected PFOA and parity-specific BW-for-GA *z*-scores and sex-specific BW-for-GA z-scores remained unchanged from single pollutant to multipollutant models, although statistical significance was only reached in the parity-specific model (β= −0.3; 95% CI −0.6, −0.01). PFNA, PFDA, PFOS, and PFHxS were not associated with any birthweight measure in multipollutant models. The results are shown in Table 4.

**Table 4:** Linear regression model results for five PFAS compounds simultaneously on continuous infant birthweight and sex– or parity–specific BW–for–GA *z*–scores (*n*, 342).

**PFAS**	**Birthweight^a^**	**BW–for–GA *z*–scores, sex^b^**	**BW–for–GA *z*–scores, parity^c^**
	**β (95% CI)**	**β (95% CI)**	**β (95% CI)**
PFOS	29.3^§^ (−81.2, 139.7)	0.1^§^ (−0.2, 0.3)	0.1^§^ (−0.2, 0.4)
PFHxS	85.6^§^ (−48.9, 220.1)	0.2^§^ (−0.1, 0.6)	0.2^§^ (−0.1, 0.6)
PFOA (≤LOD)	REF	REF	REF
PFOA (> LOD)	−108.8 (−229.9, 12.3)	−0.3 (−0.6, 0.02)	−0.3 (−0.6, −0.01)^*^
PFNA (≤LOD)	REF	REF	REF
PFOA (> LOD)	−57.9 (−185.9, 69.9)	−0.1 (−0.4, 0.2)	−0.1 (−0.4, 0.2)
PFDA (≤LOD)	REF	REF	REF
PFDA (>LOD)	−39.2 (−138.9, 60.4)	−0.1 (−0.4, 0.1)	−0.2 (−0.4, 0.1)

*CI, Confidence interval; PFHxS, Perfluorohexanesulfonic acid; PFOS, Perfluorooctanesulfonic acid; PFNA, Perfluorononanoic acid; PFOA, Perfluorooctanoic acid; PFDA, Perfluorodecanoic acid. Reference group: Samples <LOD; PFNA LOD, 0.02 ng/ml, PFDA LOD, 0.035 ng/ml, PFOA LOD, 0.035 ng/ml*.

a *Adjusted for maternal age, maternal race/ethnicity, maternal education, country of birth, maternal household income, recruitment site, GA at time of blood sample, pre–pregnancy BMI, pregnancy fish consumption, infant sex, GA at birth, parity*.

b *Adjusted for maternal age, maternal race/ethnicity, maternal education, country of birth, maternal household income, recruitment site, GA at time of blood sample, pre–pregnancy BMI, pregnancy fish consumption, and parity*.

c *Adjusted for maternal age, maternal race/ethnicity, maternal education, country of birth, maternal household income, recruitment site, GA at time of blood sample, pre–pregnancy BMI, pregnancy fish consumption, and infant sex, n, 331*.

§ *β-Estimate interpreted as per natural log increase*.

* *p < 0.05*.

### Sensitivity Analyses

In models that were restricted to participants without GDM, gestational hypertension, or preeclampsia/eclampsia recorded on their medical records (*n* = 261), estimates were comparable to both single pollutant and multipollutant models with the full sample (Supplementary Table S3). We also found that restricting to women with PFAS concentrations measured prior to 30 weeks of gestation (*n* = 239) did not materially change our results (Supplementary Table S4).

## Discussion

We found that women with detectable levels of PFOA during pregnancy had infants who weighed on average 120 g less than women without detectable levels of PFOA. Within models that were mutually adjusted for five detected PFAS compounds, the effect size for the associations of birthweight with PFOA remained comparable. We did not find significant adjusted associations with the remaining PFAS concentrations and the birth outcomes assessed. Additionally, many PFAS compounds analyzed within the MADRES cohort had limited to no detection.

In the MADRES cohort, median PFNA, PFOA, PFOS, and PFDA concentrations were lower in relation to several other pregnancy cohorts within Asian, European, and North American countries, although this trend was not consistent with PFHxS, which was often comparable or had higher concentration levels (38, 40, 53–55). Although we do not know why PFHxS concentrations were higher in this cohort, PFHxS did have one of the longest half-lives of PFAS compounds (5–9 years) (10, 56). The highest levels of PFAS concentrations were found in participants who were pregnant with their first-born child. This is consistent with the prior literature (57–62), as breastfeeding has been shown to be a probable excretion route for PFAS (59, 61). Prior studies have also reported higher levels of PFAS within non-Hispanic white women, although research is scarce for comparisons to Hispanic women outside NHANES. PFOA and PFHxS have been seen to be higher in non-Hispanic white women compared to Black/African-American women (63) as well as to Chinese-American and Japanese-American women (64). These results are surprising given that communities of color are disproportionately burdened by environmental chemical exposures including hazardous waste sites, air pollution, and water pollution (65). PFAS exposure sources related to race and ethnicity merit further examination.

The observation that higher education levels and annual household income were associated with higher PFAS levels within MADRES is also consistent with previous studies (58, 66, 67). Foreign-born participants having lower levels of PFOS and PFHxS as seen in this study is consistent with a previous study of middle-aged women, which found that within Chinese and Japanese women, those born outside of the USA had lower PFAS concentrations compared to those born within the USA (64).

Our findings that PFAS levels were inversely related with pre-pregnancy BMI are more complex. A previous study in Norway consisting of white and primarily of normal weight women showed prenatal PFHxS levels to be significantly positively associated with pre-pregnancy BMI (66), which is contrary to our findings. We found that PFAS levels were higher among women who reported higher intakes of seafood. This is consistent with a study among Norwegian women in which unadjusted PFAS serum levels were associated with increased dietary intakes of shellfish and oily fish (66).

The majority of studies on prenatal PFAS exposure and infant birthweight have investigated individual PFAS exposures. Steenland et al. found prenatal PFOA measured in maternal or cord blood to be associated with a decrease of 10.5 g (95% CI −16.7, −4.4) in infant birthweight/ng/ml increase in PFOA in the primary random effect meta-analysis (33). Starling et al. found that significant results are similar to those reported in this current study in 628 mother–infant pairs within the Healthy Start Study. They found that women in the highest tertile of mid-pregnancy measured PFOA exposure had infants who weighed significantly less compared to those in the lowest tertile of PFOA exposure (β = −92.4 g; 95% CI −166.2, −18.5) (55). A recent study found that prenatal PFOA measured in the first trimester serum were significantly associated with decreased infant birthweight in 1,533 participants from the Swedish Environmental, Longitudinal, Mother and child, Asthma and allergy (SELMA) study. Mothers in the highest quartile of PFOA exposure had infants who weighed 90 g less (95% CI −159, −91) compared to those in the lowest quartile (37).

In this present study, we observed no consistent significant findings between PFDA, PFNA, PFOS, and PFHxS and infant birthweight. However, we found that non-significant inverse associations was observed for prenatal PFNA, PFDA, and PFOS with birthweight and a non-significant positive association was observed with PFHxS and birthweight. Previous studies have shown significant inverse relationships between PFOS exposure and birthweight in a subset of several studies (68–72), and three other studies have found non-significant positive relationships with PFHxS and birthweight (35, 38, 70). Few studies have examined relationships between PFNA and PFDA with birthweight, although significant inverse relationships have been observed (35, 37–40). Our study did not find any significant results with PFAS and gestational age at birth and, therefore, gestational age was not considered to be a likely mediator of the association of PFAS on infant birthweight (31, 52). Several other studies have also not found significant associations between prenatal PFAS exposure and gestational age at birth (41, 53, 73).

Many important strengths are found within the current study. First, multiple PFAS exposures were analyzed with the same panel and, in particular, we had measurements of PFDA, PFNA, and PFHxS, which have not been as comprehensively studied as PFOA and PFOS. Second, PFAS concentrations were measured in maternal blood serum, which is a superior biospecimen to whole blood or urine, with the serum to whole blood ratio for PFAS being 2:1 (74, 75). Third, our study population is comprised of predominately low-income Hispanic participants, which is an understudied group. Additionally, given that pregnant persons are exposed to more than one compound at once, we also assessed associations within a multipollutant framework and found that PFOA was consistently associated with birthweight. Lastly, the prospective cohort design and high quality covariate data are additional strengths.

The limitations of this study include only one measurement of PFAS concentrations, and PFAS were measured in the serum collected across a wide range of gestational age. For these reasons, this analysis was unable to determine critical windows of exposure of PFAS on infant birthweight. As such, while we did control for gestational age at the time of collection, we assume that PFAS levels are relatively stable across pregnancy due to their long half-lives. A previous study with PFOA and PFOS blood serum levels taken from the first and second trimester found a high degree of correlation between the two time points, but the averages were lower in the second trimester (69). An additional study with blood samples taken at all three trimesters of pregnancy found PFOA, PFOS, PFNA, and PFDA to slightly decrease over pregnancy while PFHxS levels remained consistent (35). We were unable to confirm the PFAS levels at birth measured in cord blood within this study. We also did not have available information on the glomerular filtration rate (GFR), which can influence both PFAS levels later in pregnancy and infant birthweight (76) as GFR peaks during 30–35 weeks of gestation (77). In our study, we conducted a sensitivity analysis limiting our analysis to samples collected prior to 30 weeks of gestation and we found no measurable impact on our effect estimates. Additionally, although many key covariates were adjusted for final models, residual confounding is still possible, as in all observational studies.

## Conclusion

Prenatal exposure to PFOA was associated with lower birthweight in infants in the MADRES cohort in Los Angeles, CA. These findings suggest that exposure to these chemicals during *in utero* development may have important implications for children's health. While we found the highest exposures to PFAS among non-Hispanic white women as well as women with higher household incomes and higher attained education levels in our cohort, future studies should focus on how mixtures of newer PFAS, especially those that have become substitutes for legacy PFAS including PFOA and PFOS, impact populations facing disparities in birth and other health outcomes.

## Data Availability Statement

The original contributions presented in the study are included in the article/Supplementary Materials, further inquiries can be directed to the corresponding author.

## Ethics Statement

The studies involving human participants were reviewed and approved by University of Southern California Institutional Review Board (IRB). The patients/participants provided their written informed consent to participate in this study.

## Author Contributions

Conceptualization: CB, TB, and AP. Data curation: AP, DF, KK, MR, and TY. Formal analysis, validation, visualization, and writing—original draft: AP. Funding acquisition, supervision, investigation, resources, and project administration: CB and TB. Methodology: BG, CB, DW, EG, LA-M, RH, SF, SE, and TB. Software: AP and TY. Writing—review and editing: AP, SE, RH, TY, DF, SF, BG, KK, MR, DL, LA-M, DW, EG, TB, and CB. All authors contributed to the article and approved the submitted version.

## Funding

This work was supported by the MADRES Center (Grant Nos. P50ES026086, 83615801, and P50MD015705) funded by the National Institute of Environmental Health Sciences, the National Institute for Minority Health and Health Disparities and the Environmental Protection Agency; the Southern California Environmental Health Sciences Center (Grant No. 5P30ES007048) funded by the National Institute of Environmental Health Sciences, and the Life course Approach to Developmental Repercussions of Environmental Agents on Metabolic and Respiratory health (LA DREAMERs) (Grant No. UH3OD023287) funded by the National Institutes of Health Office of the Director ECHO Program. The funding agencies had no role in the design of the study, the collection, analysis, or interpretation of data or in the writing of the manuscript.

## Conflict of Interest

The authors declare that the research was conducted in the absence of any commercial or financial relationships that could be construed as a potential conflict of interest.

## Publisher's Note

All claims expressed in this article are solely those of the authors and do not necessarily represent those of their affiliated organizations, or those of the publisher, the editors and the reviewers. Any product that may be evaluated in this article, or claim that may be made by its manufacturer, is not guaranteed or endorsed by the publisher.
