# Supplementary material for: Prenatal Perfluorooctanoic Acid (PFOA) Exposure Is Associated With Lower Infant Birthweight Within the MADRES Pregnancy Cohort
Source: Front Epidemiol. 2022 Jul 13;2:934715. doi: 10.3389/fepid.2022.934715 (PMC10910958; doi:10.3389/fepid.2022.934715)
Supplement: Supplementary file 1 [file Table_1.DOCX]

Supplementary Material

# Supplementary Figures and Tables

Figure 1. Directed Acyclic Graph (DAG) of PFAS Exposure and Infant Birthweight


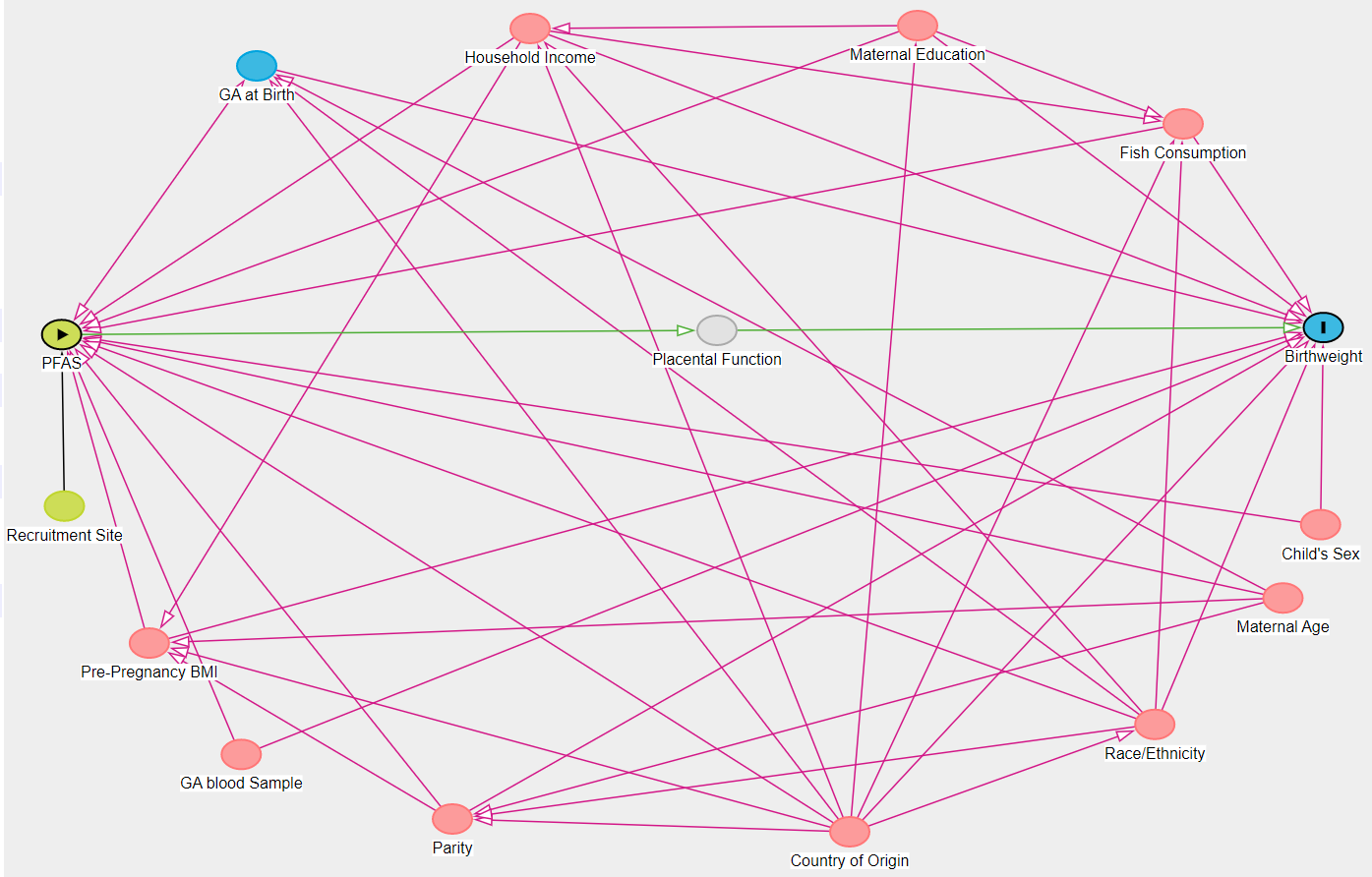


Note: Diagram was visualized using DAGitty software.

Table 1. Median PFAS Analyte Concentrations (ng/mL) by Maternal Demographic Characteristics

|  | PFOS | PFHxS | PFOA | PFDA | PFNA |
| --- | --- | --- | --- | --- | --- |
| Race/Ethnicity  Hispanic (n=260)  Non-Hispanic Black (n=37)  Non-Hispanic white (n=30)  Non-Hispanic other (n=15) | 1.27^**^  1.45^**^  1.95^**^  1.76^**^ | 1.01^**^  1.08^**^  1.81^**^  1.34^**^ | 0.11^**^  0.05^**^  0.52^**^  0.35^**^ | 0.04  0.04  0.05  0.08 | 0.07^*^  0.06^*^  0.16^*^  0.14^*^ |
| Household Income  <$50,000 (n=165)  $50,000-$99,999 (n=65)  >$100,000 (n=28)  Reported “Don’t Know” (n=84) | 1.27^**^  1.48^**^  1.99^**^  1.26^**^ | 1.05^**^  0.98^**^  1.69^**^  1.13^**^ | 0.10^**^  0.19^**^  0.59^**^  0.06^**^ | 0.05  0.04  0.07  0.04 | 0.06^**^  0.11^**^  0.23^**^  0.06^**^ |
| Education  Completed high school or less (n=181)  Some college of completed college (n=133)  Some graduate training (n=28) | 1.26^**^  1.38^**^  2.06^**^ | 1.00^**^  1.15^**^  1.49^**^ | 0.08^**^  0.12^**^  0.53^**^ | 0.04  0.04  0.05 | 0.06^**^  0.09^**^  0.21^**^ |
| Country of Birth  USA (n=175)  Other (n=157)  Unknown (n=10) | 1.44^*^  1.26^*^  1.08^*^ | 1.13^*^  1.01^*^  1.30^*^ | 1.18  0.09  0.10 | 0.04  0.05  0.03 | 0.09  0.07  0.06 |
| Birth Order of Child  First Born (n=119)  Second or more born (n=212)  Unknown (n=11) | 1.63^**^  1.25^**^  1.21^**^ | 1.25^**^  0.99^**^  1.30^**^ | 0.35^**^  0.07^**^  0.10^**^ | 0.05^*^  0.04^*^  0.05^*^ | 0.16^**^  0.06^**^  0.07^**^ |
| Pregnancy Fish Consumption  Never (n=214)  Monthly (n=55)  At least Weekly (n=42)  Unknown (n=31) | 1.26^*^  1.43^*^  1.73^*^  1.38^*^ | 1.07^*^  1.19^*^  1.28^*^  0.90^*^ | 0.09  0.18  0.23  0.22 | 0.04  0.04  0.07  0.02 | 0.06^*^  0.11^*^  0.14^*^  0.07^*^ |

_*_Kruskal-Wallis One Way ANOVA p<0.05 _**_Kruskal-Wallis One Way ANOVA p<0.0001

Supplemental Table 2. Univariate and Adjusted Models for Independent Prenatal PFAS Compounds and Gestational Age at Birth, in Weeks (N=342)

| Compound | Unadjusted  $\beta$ (95% CI) | Adjusted*  $\beta$ (95% CI) |
| --- | --- | --- |
| PFHxS  < 0.79 ng/mL  0.79- 1.08 ng/mL  1.09-1.46 ng/mL  $\geq$1.47 ng/mL | 0.3^1^ (-0.1, 0.8)  REF  -0.1 (-0.6, 0.5)  -0.1 (-0.6, 0.5)  0.4 (-0.2, 0.9) | 0.1^1^ (-0.5, 0.6)  REF  -0.03 (-0.5, 0.5)  -0.3 (-0.8, 0.3)  0.2 (-0.5, 0.5) |
| PFOS  <0.97 ng/mL  0.97-1.33 ng/mL  1.34-1.85 ng/mL  $\geq$1.86 ng/mL | 0.2^1^ (-0.2, 0.5)  REF  0.3 (-0.3, 0.8)  0.003 (-0.5, 0.5)  0.1 (-0.4, 0.7) | -0.3^1^ (-0.4, 0.3)  REF   - 1. (-0.3, 0.7)   -0.1 (-0.6, 0.4)  -0.2 (-0.7, 0.4) |
| PFDA  $\leq$LOD  >LOD | REF  0.1 (-0.3, 0.5) | REF  0.02 (-0.3, 0.4) |
| PFNA  $\leq$LOD  >LOD | REF  -0.1 (-0.5, 0.3) | REF  -0.3 (-0.7, 0.1) |
| PFOA  $\leq$LOD  >LOD | REF  0.002 (-0.4, 0.4) | REF  -0.1 (-0.5, 0.3) |

Note: CI=confidence interval; PFHxS=Perfluorohexanesulfonic acid; PFOS=Perfluorooctanesulfonic acid; PFNA=Perfluorononanoic acid; PFOA=Perfluorooctanoic acid; PFDA=Perfluorodecanoic acid. Reference group: Samples <LOD PFNA LOD=0.02 ng/mL, PFDA LOD=0.035 ng/mL, PFOA LOD=0.035 ng/mL.

^*^Adjusted for maternal age, pre-pregnancy BMI, maternal education, country of birth, household income, mother’s race, gestational age at time of blood sample, study recruitment site, parity, infant sex, and fish consumption
^1^Beta estimate represents a per natural log increase

Table 3. Maternal Serum PFAS and Birthweight Models Restricted to Participants Without Preeclampsia/Eclampsia, Gestational Hypertension or Gestational Diabetes Mellitus (GDM) (N=261)

| Model | Birthweight^1^  $\beta$ (95% CI) | BW-for-GA, sex^2^  $\beta$ (95% CI) | BW-for-GA, parity^3^  $\beta$ (95% CI) |
| --- | --- | --- | --- |
| **Single Pollutant Models** |  |  |  |
| PFOS  <0.97 ng/mL  0.97-1.33 ng/mL  1.34-1.85 ng/mL  $\geq$1.86 ng/mL | -17.5^§^ (-120.3, 85.2)  REF  -41.5(-187.9, 104.9)  -24.4 (-169.8, 120.9)  -22.2 (-176.7, 132.3) | -0.1^§^ (-0.3, 0.2)  REF  -0.1 (-0.5, 0.2)  -0.1 (-0.5, 0.3)  -0.1 (-0.5, 0.3) | -0.1^§^ (-0.3, 0.2)  REF  -0.2^§^ (-0.6, 0.2)  -0.1 (-0.5, 0.3)  -0.2 (-05, 0.2) |
| PFHxS  < 0.79 ng/mL  0.79- 1.08 ng/mL  1.09-1.46 ng/mL  $\geq$1.47 ng/mL | 106.6^§^ (-48.1, 261.2)  REF  93.8 (-50.4, 238.0)  77.4 (-85.5, 240.4)  190.7 (14.6, 366.8)* | 0.3^§^ (-0.1, 0.7)  REF  0.3 (-0.1, 0.6)  0.2 (-0.2, 0.6)  0.5 (0.02, 0.9)* | 0.3^§^ (-0.1, 0.7)  REF  0.3 (-0.1, 0.6)  0.2 (-0.2, 0.6)  0.5 (0.01, 0.9)* |
| PFOA  $\leq$LOD  >LOD | REF  -121.9 (-232.9, -10.9)* | REF  -0.3 (-0.6, -0.03)* | REF  -0.3 (-0.6, -0.1)* |
| PFNA  $\leq$LOD  >LOD | REF  -114.7 (-229.2, -0.2)* | REF  -0.3 (-0.6, 0.003) | REF  -0.3 (-0.6, -0.01)* |
| PFDA  $\leq$LOD  >LOD | REF  -34.9 (-138.2, 68.3) | REF  -0.1 (-0.4, 0.1) | REF  -0.2 (-0.4, 0.1) |
| **Multipollutant Model** |  |  |  |
| PFOS  PFHxS  PFOA ($\leq$LOD)  PFOA (> LOD)  PFNA ($\leq$LOD)  PFNA (>LOD)  PFDA ($\leq$LOD)  PFDA (>LOD) | 47.3^§^ (-78.5, 173.0)  126.3^§^ (-32.3, 284.9)  REF  -107.2 (-247.8, 33.4)  REF  -81.8 (-230.8, 67.3)  REF  -21.9 (-135.6, 91.8) | 0.1^§^ (-0.2, 0.4)  0.3^§^ (-0.1, 0.7)  REF  -0.3 (-0.6, 0.1)  REF  -0.2 (-0.5, 0.2)  REF  -0.1 (-0.4, 0.2) | 0.1^§^ (-0.2, 0.5)  0.3^§^ (-0.1, 0.7)  REF  -0.3 (-0.7, 0.02)  REF  -0.2 (-0.6, 0.2)  REF  -0.1 (-0.4, 0.2) |

Note: CI= confidence interval; PFHxS=Perfluorohexanesulfonic acid; PFOS=Perfluorooctanesulfonic acid; PFNA=Perfluorononanoic acid; PFOA=Perfluorooctanoic acid; PFDA=Perfluorodecanoic acid. Reference group: Samples <LOD PFNA LOD=0.02 ng/mL, PFDA LOD=0.035 ng/mL, PFOA LOD=0.035 ng/mL. Multipollutant model is mutually adjusted for all five analytes.
^1^Adjusted for maternal race, maternal age at study recruitment, maternal education, country of birth, maternal household income, recruitment site, gestational age at time of blood sample, pre-pregnancy BMI, pregnancy fish consumption, infant sex, gestational age at birth, parity
^2^Adjusted for maternal race, maternal age at study recruitment, maternal education, country of birth, maternal household income, recruitment site, gestational age at time of blood sample, pre-pregnancy BMI, pregnancy fish consumption, and parity
^3^Adjusted for maternal race, maternal age at study recruitment, maternal education, country of birth, maternal household income, recruitment site, gestational age at time of blood sample, pre-pregnancy BMI, pregnancy fish consumption, and infant sex; N=251
*p<0.05
^§^Beta estimate interpreted as per natural log increase

Table 4. Maternal Serum PFAS and Birthweight Models Restricted to Samples Collected Prior to 30 Weeks Gestation (N=239)

| Model | Birthweight^1^  $\beta$ (95% CI) | BW-for-GA, sex^2^  $\beta$ (95% CI) | BW-for- GA, parity^3^  $\beta$ (95% CI) |
| --- | --- | --- | --- |
| **Single Pollutant Models** |  |  |  |
| PFOS  <0.97 ng/mL  0.97-1.33 ng/mL  1.34-1.85 ng/mL  $\geq$1.86 ng/mL | -53.3^§^ (-166.9, 60.2)  REF  -11.1 (-172.4, 150.2)  -48.4 (-212.0, 115.2)  -60.2 (-227.5, 107.1) | -0.1^§^ (-0.4, 0.1)  REF  -0.06 (-0.5, 0.3)  -0.1 (-0.5, 0.3)  -0.2 (-0.6, 0.3) | -0.2^§^ (-0.4, 0.1)  REF  -0.1 (-0.5, 0.3)  -0.1 (-0.5, 0.3)  -0.2 (-0.6, 0.2) |
| PFHxS  < 0.79 ng/mL  0.79- 1.08 ng/mL  1.09-1.46 ng/mL  $\geq$1.47 ng/mL | 36.7^§^ (-125.3, 198.8)  REF  71.7 (-71.7, 215.0)  15.5 (-145.5, 176.5)  119.7 (-81.3, 320.6) | 0.1^§^ (-0.3, 0.5)  REF  0.2 (-0.2, 0.5)  0.04 (-0.4, 0.4)  0.3 (-0.2, 0.8) | 0.1^§^ (-0.3, 0.5)  REF  0.2 (-0.1, 0.6)  0.1 (-0.3, 0.5)  0.3 (-0.2, 0.8) |
| PFOA  $\leq$LOD  >LOD | REF  -121.0 (-242.4, 0.3) | REF  -0.3 (-0.6, -0.003)* | REF  -0.3 (-0.6, -0.007)* |
| PFNA  $\leq$LOD  >LOD | REF  -127.7 (-254.2, -1.2)* | REF  -0.3 (-0.6, -0.01)* | REF  -0.3 (-0.7, -0.03)* |
| PFDA  $\leq$LOD  >LOD | REF  -85.9 (-196.4, 24.6) | REF  -0.2 (-0.5, 0.05) | REF  -0.3 (-0.5, 0.02) |
| **Multipollutant Model** |  |  |  |
| PFOS  PFHxS  PFOA ($\leq$LOD)  PFOA (> LOD)  PFNA ($\leq$LOD)  PFNA (>LOD)  PFDA ($\leq$LOD)  PFDA (>LOD) | 44.5^§^ (-110.1, 199.1)  69.3^§^ (-103.1, 241.6)  REF  -97.5 (-258.2, 63.3)  REF  -77.9 (-245.3, 89.4)  REF  -67.4 (-192.0, 57.3) | 0.1^§^ (-0.3, 0.5)  0.2^§^ (-0.2, 0.6)  REF  -0.2 (-0.6, 0.2)  REF  -0.2 (-0.6, 0.2)  REF  -0.2 (-0.5, 0.1) | 0.1^§^ (-0.3, 0.5)  0.2^§^ (-0.2, 0.7)  REF  -0.2 (-0.6, 0.2)  REF  -0.2 (-0.5, 0.1)  REF  -2 (-0.5, 0.1) |

Note: CI= confidence interval; PFHxS=Perfluorohexanesulfonic acid; PFOS=Perfluorooctanesulfonic acid; PFNA=Perfluorononanoic acid, PFOA = Perfluorooctanoic acid; PFDA=Perfluorodecanoic acid. Reference group: Samples <LOD PFNA LOD=0.02 ng/mL, PFDA LOD=0.035 ng/mL, PFOA LOD=0.035 ng/mL. Multipollutant model is mutually adjusted for all five analytes.
^1^Adjusted for maternal race, maternal age at study recruitment, maternal education, country of birth, maternal household income, recruitment site, gestational age at time of blood sample, pre-pregnancy BMI, pregnancy fish consumption, infant sex, gestational age at birth, parity
^2^Adjusted for maternal race, maternal age at study recruitment, maternal education, country of birth, maternal household income, recruitment site, gestational age at time of blood sample, pre-pregnancy BMI, pregnancy fish consumption, and parity
^3^Adjusted for maternal race, maternal age at study recruitment, maternal education, country of birth, maternal household income, recruitment site, gestational age at time of blood sample, pre-pregnancy BMI, pregnancy fish consumption, and infant sex
*p<0.05
^§^Beta estimate interpreted as per natural log increase
